# Prioritizing susceptibility genes for the prognosis of male-pattern baldness with transcriptome-wide association study

**DOI:** 10.1186/s40246-024-00591-y

**Published:** 2024-04-02

**Authors:** Eunyoung Choi, Jaeseung Song, Yubin Lee, Yeonbin Jeong, Wonhee Jang

**Affiliations:** Department of Life Sciences, Dongguk University, Seoul, 04620 Republic of Korea

**Keywords:** Drug repositioning, Gene prioritization, Male-pattern baldness, Phenome-wide association study, Transcriptome-wide association study

## Abstract

**Background:**

Male-pattern baldness (MPB) is the most common cause of hair loss in men. It can be categorized into three types: type 2 (T2), type 3 (T3), and type 4 (T4), with type 1 (T1) being considered normal. Although various MPB-associated genetic variants have been suggested, a comprehensive study for linking these variants to gene expression regulation has not been performed to the best of our knowledge.

**Results:**

In this study, we prioritized MPB-related tissue panels using tissue-specific enrichment analysis and utilized single-tissue panels from genotype-tissue expression version 8, as well as cross-tissue panels from context-specific genetics. Through a transcriptome-wide association study and colocalization analysis, we identified 52, 75, and 144 MPB associations for T2, T3, and T4, respectively. To assess the causality of MPB genes, we performed a conditional and joint analysis, which revealed 10, 11, and 54 putative causality genes for T2, T3, and T4, respectively. Finally, we conducted drug repositioning and identified potential drug candidates that are connected to MPB-associated genes.

**Conclusions:**

Overall, through an integrative analysis of gene expression and genotype data, we have identified robust MPB susceptibility genes that may help uncover the underlying molecular mechanisms and the novel drug candidates that may alleviate MPB.

**Supplementary Information:**

The online version contains supplementary material available at 10.1186/s40246-024-00591-y.

## Introduction

Male-pattern baldness (MPB), also known as androgenetic alopecia, is the leading cause of hair loss in men. MPB is a polygenic disease with a high heritability rate (~ 80%), reaching a prevalence of approximately 50% by the age of 50 [1, 2]. MPB is usually caused by testosterone, an androgenic hormone, with the hairline and crown being more susceptible to androgen than the occipital scalp [3, 4]. As a result, MPB typically starts with a receding hairline and hair loss on the crown, eventually leaving only a horseshoe-shaped area of hair on the back of the head [1, 5, 6]. The severity of MPB is assessed using the Hamilton-Norwood scale (HNS) that identifies characteristic patterns of progression [7–10]. This scale is divided into three main types ranging from type 2 (mild MPB) to type 4 (severe MPB), while type 1 is considered the normal phenotype. MPB has a complex pathophysiology, potentially involving interactions between various tissues including skin, adipose, blood, and other endocrine systems [11]. Currently, finasteride and dutasteride are used to temporarily alleviate MPB by inhibiting or decomposing testosterone, so these drugs are not effective for severe MPB, may have reproductive side effects, and often result in high recurrence rates [12, 13]. Therefore, further research on the genetic basis of MPB and its application to therapeutic investigation is necessary.

Previous studies have suggested that the *androgen receptor* (*AR*) on the X chromosome and/or the genes involved with the Wnt/β-catenin signaling pathway are causal genetic factors that promote MPB [2, 4, 14]. However, these genes cannot fully explain the high heritability and mechanisms of MPB [1]. A previous genome-wide association study (GWAS) was performed to investigate the genetic features of MPB and several risk loci with high heritability were identified [1]. Although this study identified risk loci associated with MPB, it is necessary to map these loci to specific genes to gain a deeper understanding of the underlying genomic and transcriptomic mechanisms. As observed in many GWAS, the risk loci are often found in intergenic regions, such as single nucleotide polymorphisms (SNPs) in non-coding regions. These SNPs often regulate the expression of genes located far away from them, making it challenging to directly map them to genes based on their genomic locations [15–20]. In addition, GWAS identifies linkage disequilibrium (LD) blocks of associated variants rather than specific variant-trait associations [20]. Therefore, it is difficult to discover the causal variants using GWAS risk loci. To address these issues, transcriptome-wide association study (TWAS) has been proposed as a comprehensive method that takes into account SNP-mediated gene expression based on the correlation between SNPs and gene expression levels [21]. TWAS predicts the gene expression levels associated with complex traits by calculating associations with genetic variants using expression quantitative trait loci (eQTL) panels. Recently, the eQTL panels from context-specific genetics (CONTENT), which combine tissue-shared genetic features in the regulation of gene expression, have shown improved statistical power in TWAS [22]. By utilizing the advantages of TWAS that has proven effective in investigating the pathogenesis of traits and prioritizing causal genes, this study aims to address the limitations of previous studies and provide new insights into the mechanisms of MPB.

**Fig. 1:**
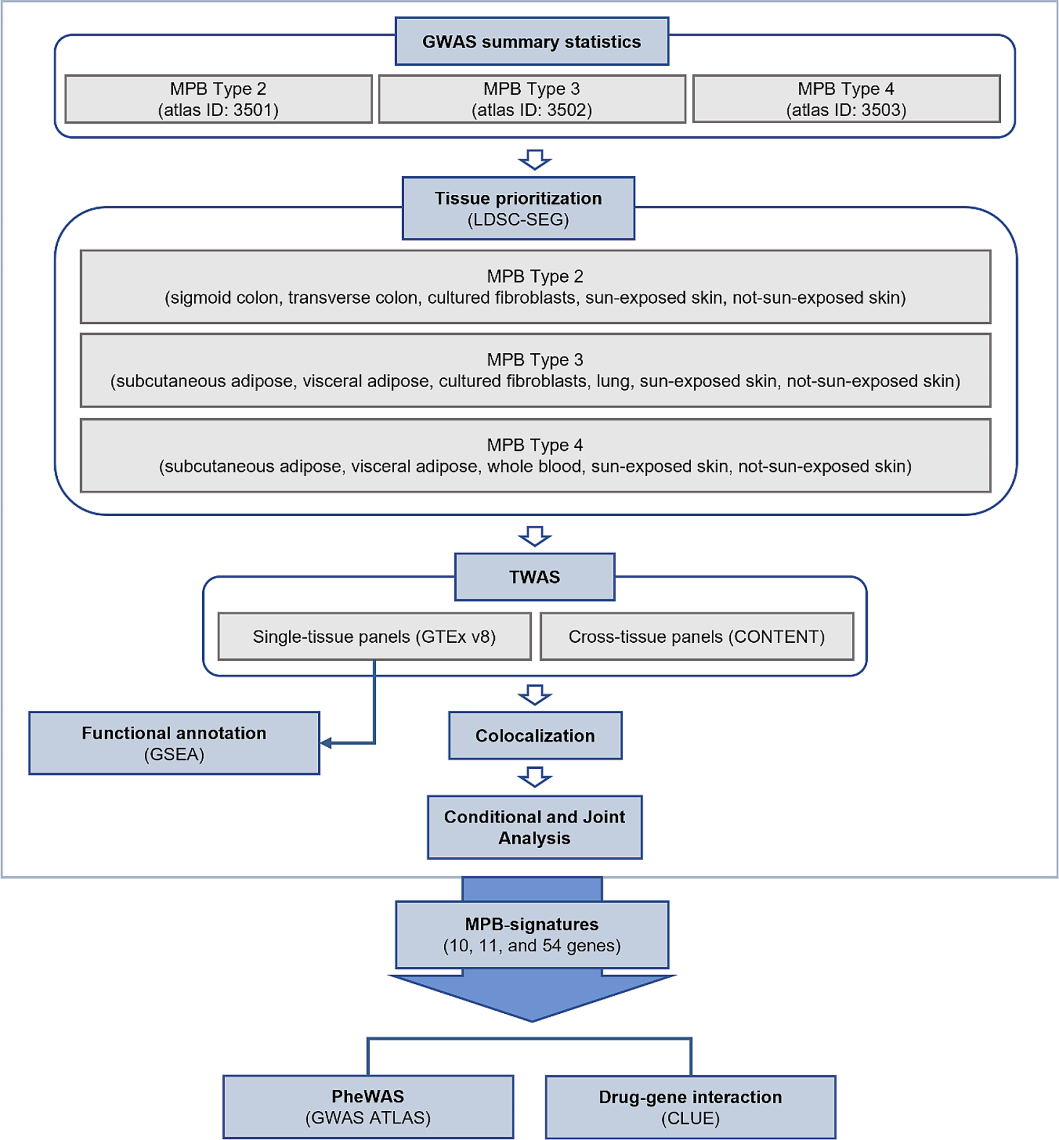
**Workflow of the entire study.** The GWAS summary statistics were obtained from GWAS ATLAS and tissue panels were prioritized using LDSC-SEG. We conducted TWAS and COLOC using both single-tissue panels and cross-tissue panels. The biological functions of TWAS results from single-tissue panels were evaluated through functional annotation analysis. Downstream analyses were performed using 10 (type 2), 11 (type 3), and 54 (type 4) genes that showed significance in the conditional and joint analysis

Here, we present a study that examines gene expression to investigate the association between risk loci and genes. We conducted TWAS on three types of MPB (type 2, type 3, and type 4) utilizing three GWAS datasets from the UK Biobank (UKBB), which consists of individuals of European ancestry. Prior to TWAS, the linkage disequilibrium score-specifically expressed genes (LDSC-SEG) method was used to prioritize tissue panels that were significantly associated with each type of MPB [23]. Using the results of the tissue prioritization with LDSC-SEG, we performed TWAS on single-tissue panels from the genotype-tissue expression (GTEx) version 8 and cross-tissue panels from the CONTENT. To verify the robustness of MPB susceptibility genes, we conducted a colocalization (COLOC) and conditional and joint analysis, which identified 10, 11, and 54 significantly associated genes with MPB type 2, type 3, and type 4, respectively. As downstream analyses of these MPB signatures, we conducted a phenome-wide association study (PheWAS) and *in silico* drug repositioning (Fig. 1).

## Materials and methods

### Description of GWAS summary statistics and data pre-processing

The GWAS summary statistics for TWAS analyses were obtained from GWAS ATLAS (https://atlas.ctglab.nl/). The datasets were categorized into four types based on the disease states of MPB: type 1 (HNS I and II), type 2 (HNS II, III, IIIa, and Iva), type 3 (HNS III vertex-V), and type 4 (HNS IV, V, Va, VI, and VII) [2, 24, 25]. Except for the normal type 1, the three datasets consisted of 176,380 Europeans from the UKBB and additional details can be found in Supplementary Table S1. The datasets were converted into the LD score format using the LD score regression (LDSC) software (version 1.0.1).

### The tissue-specific enrichment analysis based on genetic heritability

To prioritize tissue panels associated with MPB for TWAS analyses, we conducted a tissue-specific enrichment analysis using LDSC-SEG, a tool developed by Finucane et al. [23]. for identifying disease-relevant tissues. This method applies stratified LDSC to GWAS summary statistics to evaluate the contribution of the heritable element in multiple tissue types [23]. We tested the tissue-specific enrichments for the three types of MPB using the multi-tissue gene expression and multi-tissue chromatin modification (DNase hypersensitivity, histone acetylation, and histone methylation) datasets. The multi-tissue gene expression dataset consists of the GTEx [26] and Franke lab datasets [27, 28] and is classified into nine major groups for visualization purposes: adipose, blood/immune, cardiovascular, central nervous system, digestive, liver, musculoskeletal/connective, pancreas, and “other”. The multi-tissue chromatin modification dataset includes data from the Roadmap Epigenomics [29] and the Encyclopedia of DNA Elements (ENCODE) projects [30] and is classified as the multi-tissue gene expression dataset. Since chromatin modification can influence gene expression, we used the multi-tissue chromatin modification dataset to validate the results obtained from the multi-tissue gene expression dataset and to account for epigenetic modifications. While MPB is known to be associated with the sex chromosomes, we aimed to identify responses in somatic cells that occur regardless of the sex chromosomes by considering general tissues or cells rather than those specific to sex chromosomes.

### The transcriptome-wide association analysis using multi-tissue panels

The TWAS analysis was performed using the functional summary-based imputation (FUSION) software. FUSION integrates GWAS summary statistics and eQTL weights to compute gene expression-mediated associations between genotype and phenotype [21]. We used eQTL panels from GTEx version 8, which were filtered based on tissue-specific enrichment analysis. For multiple comparison adjustment, the Bonferroni correction method was applied for TWAS associations of tissue panels in MPB type 2, type 3, and type 4, respectively. To obtain additional TWAS associations with tissue-shared or tissue-specific effects, we utilized the cross-tissue expression panel from the CONTENT. The CONTENT panel computes based on the admixture of GTEx single-tissue panels and combines tissue-shared and tissue-specific associations through linear regression calculated by Thompson et al. [22] For TWAS results using the CONTENT panel, the Bonferroni correction method was used and only results corresponding to the tissue panels of tissue-specific enrichment analysis were selected.

### The colocalization analysis between eQTL and GWAS signals

Since TWAS is based on predictive models that include the genotype, gene expression, and phenotype, there may be hidden confounding factors including LD contamination, which can lead to false correlations between predicted gene expression and phenotype. To address this issue, we performed the COLOC analysis, a Bayesian-based statistical method that estimates the probability of shared causal variants between eQTL and GWAS signals [31]. The posterior probabilities (PP) for hypotheses 0–4 were as follows: H_0_ (no causal variant), H_1_ (only causal variants between genotype and phenotype), H_2_ (only causal variants for eQTL), H_3_ (phenotype and gene expressions driven by two independent causal variants), and H_4_ (phenotype and gene expressions share one causal variant). Following previous studies, we set the significance threshold as PP3 + PP4 > 0.8 and PP4 / PP3 > 2 [16, 32]. 

### The conditional and joint analysis

To ensure accuracy in identifying potential LD-contaminated associations, a conditional and joint analysis was conducted. This analysis takes into account the extent to which GWAS signal remains significant when other associations are included as covariates. If a GWAS signal remains significant even after conditioning, it suggests that the signal is conditionally independent, meaning it is not reliant on other associations. However, if the signal becomes less significant after conditioning, it suggests that the signal was influenced by other associations [15, 21]. Genes that still exhibit significance after conditioning were defined as jointly significant genes, while genes showing reduced significance after conditioning are considered as marginally significant genes due to co-regulation. We selected the jointly significant genes as the reliable genetic signature for each type of MPB and named them as MPB signatures.

### The functional enrichment analysis in biological processes using GSEA

The functional annotations of TWAS associations were conducted using the gene set enrichment analysis (GSEA) version 4.3.2, a computational method that interprets groups of genes that share a common biological function by using gene expression data and a gene set database [33]. The functional enrichment analysis utilized gene sets derived from the gene ontology (GO) biological process and ranked gene lists with z-scores calculated from TWAS. Biological processes that met a significance threshold (P-value < 0.05) were shown based on the normalized enrichment scores (NES). The NES value reflects the degree of up-regulated and down-regulated gene enrichment through multiple hypothesis testing.

### The phenome-wide association study using MPB signatures to identify MPB-associated traits

The PheWAS is a method used to search for phenotypes that share specific SNP associations across thousands of human phenotypes. This approach tests associations between genetic variants and a large number of phenotypes to discover pleiotropic effects, where specific genetic variants may be associated with multiple traits [34, 35]. By utilizing the PheWAS database available at GWAS ATLAS that contains leading SNPs from 4,756 GWAS summary statistics, we examined the phenotype associations of MPB signatures [36]. For each SNP in the MPB signatures, we obtained a list of associations and filtered the results to identify significant traits linked to genetic loci of each type of MPB using a Bonferroni-corrected significance threshold (P-value < 1.05 × 10^− 5^).

### Drug-gene connectivity analysis for drug repositioning

To identify drugs that are associated with genes, we used the connectivity map (CMAP) and library of integrated network-based cellular signatures (LINCS) unified environment (CLUE) (https://clue.io/about) that is a web-based tool for drug repositioning. CLUE generates drug-gene connections by analyzing gene expression and proteomics assays including the LINCS L1000 and CMAP databases [37, 38]. To validate the drug candidates identified by CLUE, we also used the Networkanalyst that can offer comprehensive list of protein-drug interaction networks collected from the DrugBank database (version 5.0) [39]. The networks were visualized using Cytoscape application (version 3.9.1) and only networks that were directly linked to MPB signatures in both results were displayed [40]. 

### Calculating the protein-protein interaction score

The protein-protein interaction (PPI) analysis was conducted between the already known markers and signatures of MPB. For the known MPB genes, we selected 336 genes that appeared when searching for ‘androgenetic alopecia’ in the Open Targets Platform (https://platform.opentargets.org/). The interaction score was calculated using the combined score from the STRING database (https://string-db.org/). As a control, we calculated the interaction score between randomly selected genes and known MPB genes. To determine whether the two groups of interaction scores were significantly different, we analyzed the degree of interaction using a one-tailed t-test with 10,000 repetitions.

## Results

### Panel prioritization to select MPB-related tissue panels

**Fig. 2:**
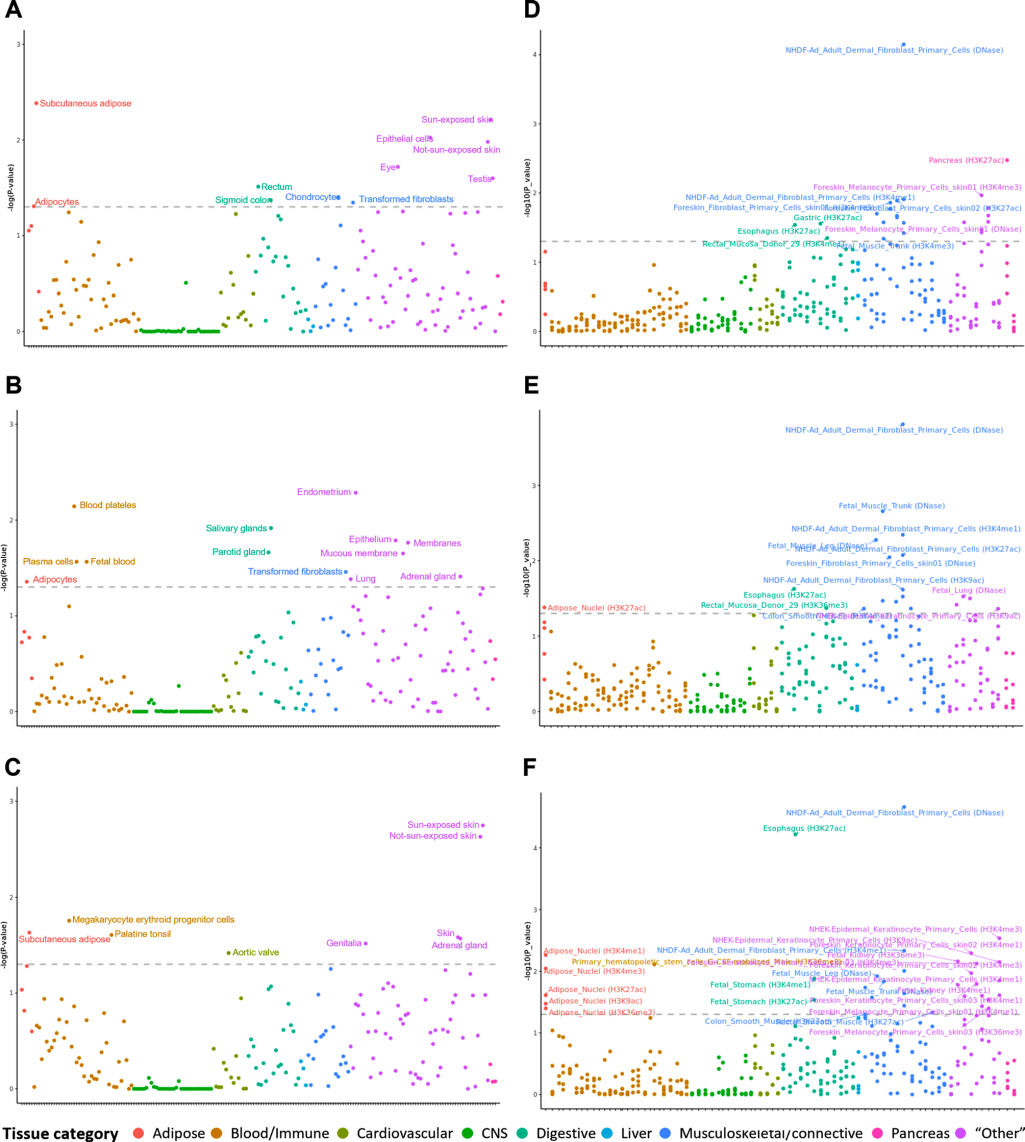
**Results of tissue prioritization using the multi-tissue gene expression and chromatin modification datasets.** Scatter plots showing the tissue prioritization of MPB type 2, 3, and 4 using both multi-tissue gene expression dataset **(A–C)** and multi-tissue chromatin modification dataset **(D–F)**. These tissue prioritization results were categorized into nine major groups and are plotted on the X-axis (scarlet: adipose; mustard: blood/immune; yellow green: cardiovascular; green: central nervous system; blue green: digestive; turquoise: liver; blue: musculoskeletal/connective; pink: pancreas; and lilac: “other”). The Y-axis represents –log(P-value) and the dashed lines indicate the significant thresholds (coefficient P-value < 0.05). Any tissues above the threshold are considered significant

Since TWAS calculates genetic associations based on tissue panels, it is necessary to select appropriate tissues to obtain accurate results. In particular, to determine the effects of MPB on somatic cells, it is better to select MPB-related tissue panels from general tissue panels rather than focusing on tissue panels related to the sex chromosomes. To examine the genetic contribution in tissue-specific gene expression of MPB and select significantly associated tissue panels, we conducted a tissue-specific enrichment analysis using the LDSC-SEG with the multi-tissue gene expression dataset [23]. The LDSC-SEG analysis with the multi-tissue gene expression dataset suggested that four (adipose, digestive, musculoskeletal/connective, and “other”) out of nine major categories showed significant association with MPB type 2 (coefficient P-value < 0.05) (Fig. 2A). For MPB type 3, we identified significant enrichment in five (adipose, blood/immune, digestive, musculoskeletal/connective, and “other”) out of nine major categories (coefficient P-value < 0.05) (Fig. 2B). In the case of MPB type 4, the analysis revealed significant associations in four major categories (adipose, blood/immune, cardiovascular, and “other”; coefficient P-value < 0.05) (Fig. 2C and Supplementary Data S1).

To validate the results of the tissue-specific enrichment analysis using the multi-tissue gene expression dataset, we also utilized the multi-tissue chromatin modification dataset [23]. For MPB type 2, the analysis revealed significant enrichment in four (digestive, musculoskeletal/connective, pancreas, and “other”) out of nine major categories, of which three categories showed significance in both the multi-tissue gene expression and multi-tissue chromatin modification datasets (coefficient P-value < 0.05) (Fig. 2D). The results for MPB type 3 showed significant enrichment in four major categories (adipose, digestive, musculoskeletal/connective, and “other”; coefficient P-value < 0.05) (Fig. 2E). These four categories were also significantly associated with MPB type 3 using the multi-tissue gene expression dataset. MPB type 4 showed significant enrichment in the adipose, blood/immune, digestive, musculoskeletal/connective, and “other” categories, three of which showed significance in both the multi-tissue gene expression and multi-tissue chromatin modification datasets (coefficient P-value < 0.05) (Fig. 2F). The specific tissue categories and statistics for MPB type 2, type 3, and type 4 are presented in Supplementary Data S1 and S2. Finally, we selected five, six, and five tissue eQTL panels from GTEx, based on their significant enrichment in both the multi-tissue gene expression and multi-tissue chromatin modification datasets (Table 1). These selected tissue eQTL panels were used for subsequent TWAS analyses.

**Table 1:** Results of tissue prioritization for TWAS analyses

Trait	GTEx eQTL panel
MPB Type 2	Sigmoid colon, Transverse colon, Cultured fibroblasts, Sun-exposed skin, Not-sun-exposed skin
MPB Type 3	Subcutaneous adipose, Visceral adipose, Cultured fibroblasts, Lung, Sun-exposed skin, Not-sun-exposed skin
MPB Type 4	Subcutaneous adipose, Visceral adipose, Whole blood, Sun-exposed skin, Not-sun-exposed skin

### The transcriptome-wide association study to identify associations with MPB

**Fig. 3:**
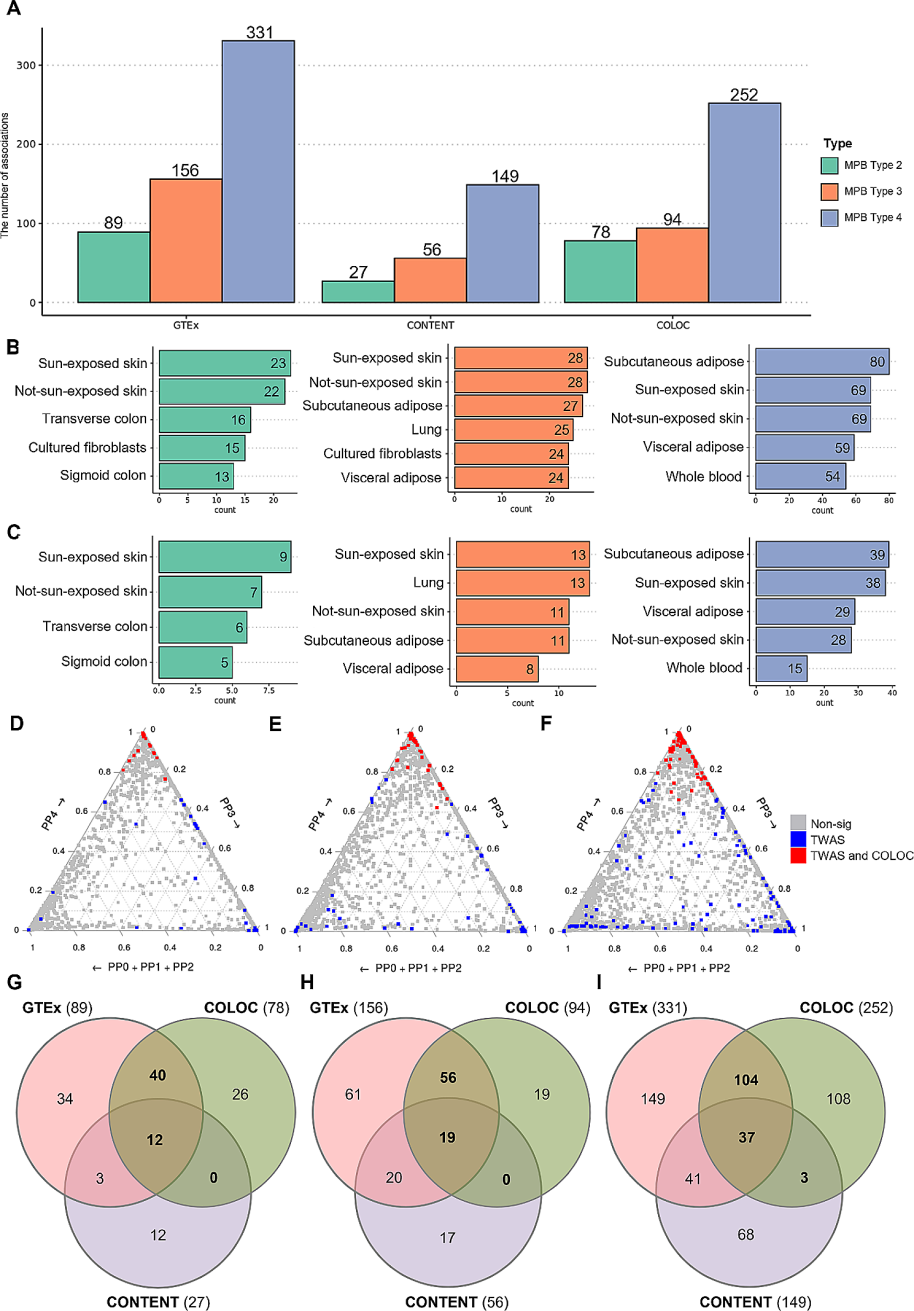
**Results of TWAS and COLOC analyses.(A)** A bar graph displaying the number of associations obtained using TWAS (GTEx and CONTENT) and COLOC, which passed the threshold for each analysis. The results are represented by different colors: green (type 2), orange (type 3), and blue (type 4). **(B)** Bar graphs showing the results of TWAS using individual tissue panels of GTEx. **(C)** Bar graphs displaying the results of TWAS using individual tissue panels of CONTENT. The bars are color-coded according to MPB types. Ternary plots showing TWAS and COLOC results for **(D)** MPB type 2, **(E)** type 3, and **(F)** type 4, respectively. PP0 + PP1 + PP2 indicates underpowered signals; PP3 indicates independent variants between phenotype and gene expression; and PP4 indicates colocalized variants where the causal SNP is associated with both phenotype and gene expression. Non-significant TWAS associations are represented with gray points, while significant associations are marked in blue, of which with a high posterior probability of colocalized signals are highlighted in red. Venn diagrams represent the number of associations from GTEx, CONTENT, and COLOC for **(G)** MPB type 2, **(H)** type 3, and **(I)** type 4, respectively. Genes used for further analysis are indicated in bold

To investigate the susceptibility genes for MPB, we conducted TWAS using the FUSION method, which identifies associations between GWAS phenotype and gene expressions. We used three GWAS summary statistics and relevant tissue eQTL panels from the GTEx selected based on tissue-specific enrichment analysis (Table 1 and Supplementary Table S1). Among a total of associations, 89, 156, and 331 associations in 20, 25, and 101 loci were significantly associated with MPB type 2, type 3, and type 4, respectively (Bonferroni-corrected P-value < 0.05) (Fig. 3A, Supplementary Fig. 1, and Supplementary Data S3). The significant associations can be divided into positive associations and negative associations based on the direction of the z-score, indicating that increased/decreased expression leads to increased/decreased risk of MPB. In MPB type 2, there were 18 positive associations and 71 negative associations. In case of MPB type 3, it had 122 positive associations and 34 negative associations, while MPB type 4 showed 182 positive associations and 149 negative associations. The number of significant associations according to tissue panels is shown in Fig. 3B. For MPB type 2 and type 3, the sun-exposed and not-sun-exposed skin panels had the largest number of associations. In the case of MPB type 4, subcutaneous adipose tissue had the largest number of associations, surpassing both skin tissue panels (Fig. 3B). Among the results, we found that 7, 10, 16 genes were significant in all tissue panels (Supplementary Fig. 2D–F). We then examined the Chi-squared TWAS z-scores for each tissue and observed that the tissue panels within individual types of MPB had similarly grouped means of effect sizes (type 2: 5.39 to 5.84, type 3: 7.70 to 8.47, and type 4: 8.57 to 9.21) (Supplementary Fig. 2A–C) [41]. This indicates that the associations of MPB may evenly affect gene expression in the tissue panels.

To examine the tissue-specific or tissue-shared genetic effects in TWAS associations at the pathway level, we performed the functional annotation for each tissue panel using GSEA. We found that all the 79, 111, and 116 pathways that were statistically significant were enriched in multiple tissue panels (Supplementary Fig. 3 and Supplementary Data S4). The majority of tissue panels showed significant enrichments in pathways previously implicated in MPB pathogenesis, including apoptosis, signaling pathway, and immune response (Supplementary Table S2) [42–45]. This suggests that the cumulative functional characteristics of TWAS associations for MPB are consistent across tissue panels and have shared effects across tissues.

Although TWAS using GTEx eQTL panels can identify associations for each tissue, it does not account for all intra-individual correlations found in multiple contexts [22]. To analyze the tissue-shared effects of MPB-associated genetic variants, we performed additional TWAS analyses using cross-tissue panels called CONTENT. Since there was no cultured fibroblast panel in CONTENT, we conducted the analysis using the remaining panels. After Bonferroni correction, we found 27, 56, and 149 associations in 6, 10, and 33 loci that were significantly associated with MPB type 2, type 3, and type 4. Among these associations, 12, 17, and 71 associations were not found in the results using GTEx panels (Bonferroni-corrected P-value < 0.05) (Supplementary Data S5, Fig. 3A and G-I). The majority of TWAS associations for GTEx and CONTENT were located on chromosome 17 that is known to be related to various human genetic diseases, including cancers, DNA damage response, and MPB (Supplementary Fig. 1) [46, 47]. Detailed results on the number of associations for each tissue panel are presented in Fig. 3C. The number of significant associations across all tissue panels was two, five, and six (Supplementary Fig. 4D–F). We also examined Pearson’s correlation between the results from the GTEx and CONTENT panels, which showed moderate to high levels of consistency with minor differences (0.62 to 0.75 of Pearson’s R). These results indicate that our analysis accounted for both tissue-specific and tissue-shared features of MPB (Supplementary Fig. 4A–C).

To filter out LD-contaminated associations and ensure rigorousness, we additionally conducted the COLOC analysis. The COLOC analysis evaluates the consistency between GWAS signals and eQTL to determine if the signals are driven by a shared causal variant by calculating PPs for hypotheses of colocalized patterns between GWAS and eQTL signals. Out of the significant associations identified in the TWAS analyses, 52, 75, and 144 associations met the COLOC threshold (PP3 + PP4 > 0.8 and PP4 / PP3 > 2) (Fig. 3D–I and Supplementary Table S3).

### The conditional and joint analysis to identify conditionally independent genes

**Fig. 4:**
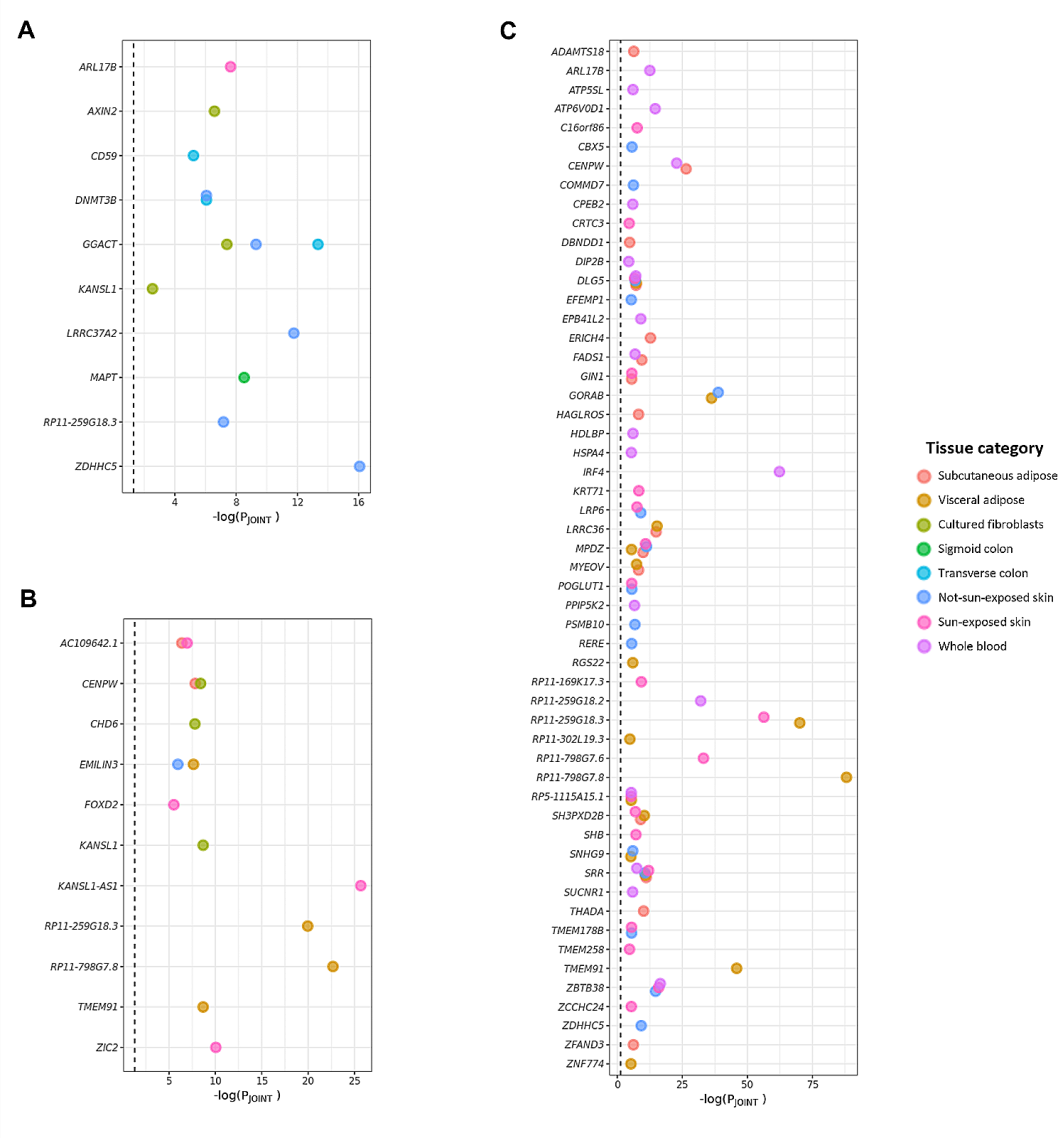
**Results of the conditional and joint analysis.** Dot plots showing joint genes that remained significant after conditioning in **(A)** MPB type 2, **(B)** type 3, and **(C)** type 4. The X-axis represents –log(joint P-value) and the dashed line indicates the significance threshold (joint P-value < 0.05). The dot color corresponds to the tissue panels (scarlet: subcutaneous adipose; mustard: visceral adipose; yellow green: cultured fibroblasts; green: sigmoid colon; light blue: transverse colon; blue: not-sun-exposed skin; pink: sun-exposed skin; and lilac: whole blood)

To thoroughly eliminate any remaining potential LD contaminations, the conditional and joint analysis were performed to determine the conditional or joint associations of significant levels [21]. We tested the joint significance of 52, 75, and 144 associations by conditioning on the GWAS signals and identified 13, 14, and 82 jointly significant associations in 9, 8, and 44 loci for MPB type 2, type 3, and type 4, respectively (joint P-value < 0.05) (Fig. 4 and Supplementary Table S4).

There were 10, 11, and 54 genes found in jointly significant associations, which were denoted as MPB signatures below, containing one, four, and nine non-coding RNAs, as well as 9, 7, and 45 protein-coding genes. Especially, among the protein coding genes, we identified two, two, and four novel genes that had not been recognized as MPB risk genes in previous studies and gene databases: *cluster of differentiation 59* (*CD59*) and *zinc finger DHHC-type palmitoyltransferase 5* (*ZDHHC5*) in MPB type 2; *chromodomain helicase DNA binding protein 6* (*CHD6*) and *zic family member 2* (*ZIC2*) in MPB type 3; and *ADAM metallopeptidase with thrombospondin type 1 motif 1*8 (*ADAMTS18*), *ATPase H + transporting V0 subunit D1* (*ATP6V0D1*), *transmembrane protein 178B* (*TMEM178B*), and *ZDHHC5* in MPB type 4 (Table 2).

**Table 2:** Novel protein-coding genes in the results of conditional and joint analysis

Trait	Gene	Z (TWAS)	P (TWAS)	Z (Joint)	P (Joint)	Tissue panel
MPB Type 2	*CD59*	-4.5	6.10E-06	-4.5	6.10E-06	Transverse colon
	*ZDHHC5*	8.3	8.60E-17	8.3	8.6E-17	Not-sun-exposed skin
MPB Type 3	*CHD6*	-5.6	1.60E-08	-5.6	1.60E-08	Cultured fibroblasts
	*ZIC2*	-6.5	8.90E-11	-6.5	8.90E-11	Sun-exposed skin
MPB Type 4	*ADAMTS18*	5.0	4.50E-07	5.0	4.50E-07	Subcutaneous adipose
	*ATP6V0D1*	-7.9	2.60E-15	-7.9	2.60E-15	Whole blood
	*TMEM178B*	-4.7	3.20E-06	-4.7	3.20E-06	Not-sun-exposed skin
		-4.7	3.20E-06	-4.7	3.20E-06	Sun-exposed skin
	*ZDHHC5*	-6.2	6.30E-10	-6.2	6.20E-10	Not-sun-exposed skin

### Phenome-wide association study of MPB signatures to identify potential pleiotropic effects

**Fig. 5:**
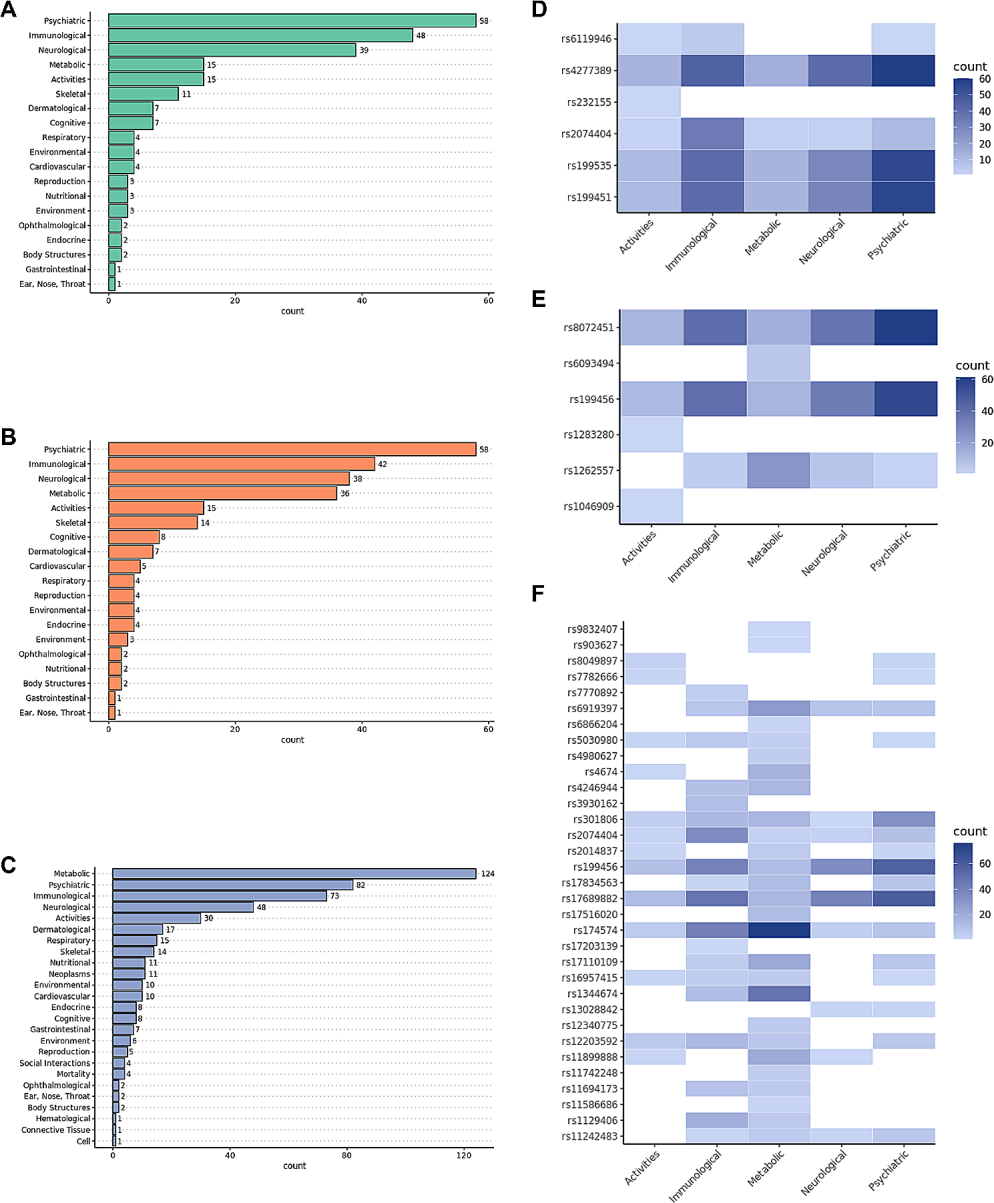
**PheWAS results of SNP-trait associations.** Bar graphs indicating the number of traits per each domain in **(A)** MPB type 2, **(B)** type 3, and **(C)** type 4. Heatmaps showing the number of traits associated with each MPB signature variant for the top five domains in **(D)** MPB type 2, **(E)** type 3, and **(F)** type 4

To explore the potential pleiotropic effects of our MPB signatures and identify shared genetic factors with other diseases or traits, we conducted a PheWAS analysis using GWAS ATLAS, which can reveal traits associated with MPB-signatures. We identified 223, 244, and 490 traits that were categorized into 19, 19, and 25 domains, respectively, under the Bonferroni-corrected significance threshold (Fig. 5A–C and Supplementary Data S6). The psychiatric domain showed the highest number of traits associated with MPB type 2 and type 3, while the metabolic domain had the highest number of traits in MPB type 4. The analysis revealed that variants from MPB signatures were highly enriched in five domains across all types of MPB: activities, immunological, metabolic, neurological, and psychiatric domains. It is well known that these domains are associated with an increased risk of MPB [1, 44, 48–51]. 

Among the variants that had pleiotropic effects, there were 6, 6, and 33 variants associated with five domains for MPB type 2, type 3, and type 4, respectively (Fig. 5D–F). Among the six variants in MPB type 2, rs4277389, rs199535, and rs199451 showed a high number of associations with psychiatric and other domains, regulating genes such as *microtubule-associated protein tau* (*MAPT*), *RP11-259G18.3*, and *KAT8 regulatory NSL complex subunit 1* (*KANSL1*), which are known to be involved in neuropsychiatric functions (Fig. 5D) [52–54]. In MPB type 3, rs8072451 and rs199456 were particularly associated with the psychiatric domain, regulating *RP11-798G7.8* and *RP11-259G18.3* (Fig. 5E). Among the 33 variants associated with MPB type 4, rs174574, which regulates *fatty acid desaturase 1* (*FADS1*) known to play a crucial role in the metabolic response of fatty acids, showed the largest number of associations with the metabolism domain (Fig. 5F) [55, 56]. We sought that these variants may cause pleiotropic effects by regulating gene expression in multiple tissues. By examining the traits associated with these variants, the traits that may be related to MPB can be confirmed.

### Identification of potential drug candidates related to MPB signatures

**Fig. 6:**
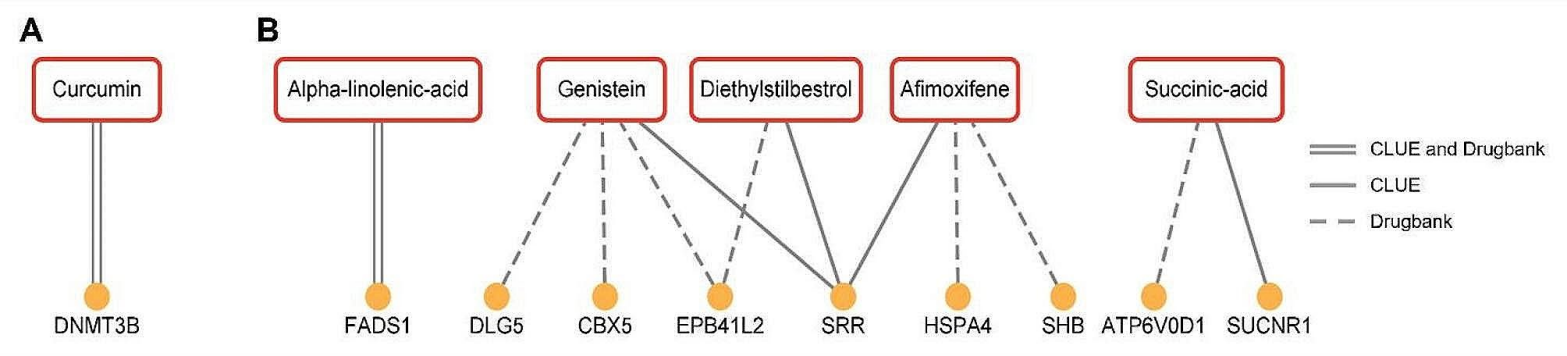
**Drug-gene interaction network using CLUE and Drugbank database.** The drug-gene interactions for **(A)** MPB type 2 and **(B)** type 4 were identified using CLUE and Drugbank database and only drugs common to both results were visualized using Cytoscape. The solid line represents drug-gene interaction from CLUE, dashed line indicates drug-gene interaction from Drugbank database, and parallel lines indicate drug-gene interaction from both CLUE and Drugbank database

In order to identify potential drug candidates for treating MPB, we analyzed the connectivity between drugs and MPB signatures using CLUE and Drugbank database. We manually excluded drugs known to cause hair loss. Among the connected drugs, curcumin was directly connected to type 2 MPB signatures (Fig. 6A). For MPB type 4, five drug molecules showed a direct connection with type 4 MPB signatures: alpha-linolenic-acid, genistein, diethylstilbestrol, afimoxifene, and succinic-acid (Fig. 6B). Previous studies on curcumin and succinic-acid have suggested their potential use in treating MPB, supporting the reliability of our potential drug candidates [57, 58]. Unfortunately, there were no drug molecules connected with type 3 MPB signatures.

Since we identified potential drug candidates based on the MPB signatures, it is important to assess the functional connectivity between MPB signatures and known MPB marker genes in order to establish credibility for our results. To address this issue, we compared the connectivity between MPB signatures and known MPB markers to the connectivity between random genes and known MPB markers (Supplementary Data S7). We calculated the interaction scores using protein interaction datasets from the STRING database. The results showed that the mean connectivity of type 2 and type 4 MPB signatures was significantly higher than that of random genes in nearly half of the cases in our simulation repeated up to 10,000 times (Supplementary Fig. 5). This indicates that MPB signatures may exhibit functional connectivity with known MPB markers, suggesting that drug candidates potentially affect the known MPB markers via MPB signatures.

## Discussion

MPB is a type of hair loss in men with specific patterns, which is caused by various genetic variants. Although previous studies have attempted to identify the MPB-associated genetic variants, the associations between genetically regulated gene expression and MPB have not been studied yet. To investigate gene expression changes influenced by genetics in MPB, we utilized TWAS, a method that calculates the expected changes in gene expression modulated by genetic variants and identifies associations between genes and the trait [16]. 

Since MPB is a complex disease that may involve multiple tissues in its development, it is crucial to prioritize tissue panels based on gene expression patterns. We conducted tissue-specific enrichment analysis using the LDSC-SEG method, utilizing multi-tissue gene expression and chromatin modification datasets. As a result, we selected five, six, and five tissue panels for MPB type 2, type 3, and type 4, respectively (Fig. 2; Table 1). These tissue panels consisted of adipose, colon, fibroblasts, skin, and whole blood. It has been reported that these tissues are associated with biological processes involved in the pathology of MPB, including inflammatory response and growth factors [59–64]. 

As indicated by our gene prioritization analyses, SNP-mediated gene expression associated with MPB exhibits both tissue-specific and tissue-shared aspects (Supplementary Fig. 3 and Fig. 3). By rigorously assessing the initial TWAS results throughout the conditional and joint analysis, we identified 10, 11, and 54 robust MPB signatures, of which two, two, and four protein-coding genes were novel, respectively (Fig. 4; Table 2). Among the type 2 MPB signatures, the *CD59* gene plays a role in defending host cells from the complement system during inflammation response, which is considered one of the causes of MPB [65, 66]. *ZDHHC5*, significant in both MPB type 2 and type 4, is involved in various biological processes, including cell adhesion, neuronal activity, and innate immune response [67–69]. In particular, reduced hair follicle cell adhesion can cause hair loss by stimulating stem cell exhaustion [70]. In MPB type 3, both the *CHD6* and *ZIC2* genes are involved with transcriptional processes by activating or repressing transcription [71, 72]. Especially, the *ZIC2* gene can inhibit Wnt/β-catenin signaling, a known cause of MPB [14, 73]. In MPB type 4, the gene product of *ADAMTS18* is a member of the ADAMTS protein family with proteolytic function [74]. Proteolytic activity affects the process of hair shedding driven by enzymatic mechanisms, which can lead to baldness with insufficient replacement of hair [75, 76]. *ATP6V0D1* is a gene encoding a component of vacuolar ATPase that utilizes ATP energy to produce a proton gradient [77]. Previous studies have shown that ATP-related processes play an important role in MPB and a previously known treatment, minoxidil, also acts on ATP processes [78–80]. *TMEM178B* is a gene that encodes a transmembrane protein and its function may be attributed to the diffusion or transport of steroid hormones including androgen [81]. Therefore, based on previous studies, these novel genes may be involved in mechanisms related to MPB.

Using these MPB signatures, we conducted PheWAS and drug repositioning as downstream analyses. The results of the PheWAS revealed that all types of MPB were commonly associated with activities, immunological, metabolic, neurological, and psychiatric domains (Fig. 5). The traits involved in the metabolic and psychiatric domains were previously reported in a GWAS study by Pirastu et al. [1] and those of activities, immunological, and neurological domains were also known to be associated with MPB [1, 2, 44, 48–51, 82–84]. One of the significantly associated traits among the activities domain was UV exposure that can cause hair damage and hair loss [82]. In the immunological domain, several immune-related cells play a significant role in hair follicle regulation and regeneration [44]. Diabetes, overweight, and high cholesterol levels were shown in the metabolism domain, which have been previously reported to be related to MPB [49, 83]. Parkinson’s disease, known as an MPB-related, has been reported to share genetic factors with MPB in previous studies, particularly in the neurological domain [2, 50]. In addition, traits related to drinking, smoking and stress in the psychiatric domain may affect the mechanisms of MPB such as hair follicle inflammation [51]. 

We analyzed the connections between genes and drug molecules to identify potential drug candidates for MPB (Fig. 6). We suggested curcumin as a potential drug candidate for MPB type 2, which have already been mentioned as potential molecules for treating baldness. Curcumin is a plant-derived substance that exhibits anti-inflammatory, anti-bacterial, and antioxidant properties. Previous studies using curcumin for the treatment of MPB have shown significant improvements in hair growth and hair loss without any side-effects [57, 85]. Additionally, curcumin analogs can block the activity of AR that plays a crucial role in MPB pathogenesis [86, 87]. For MPB type 4, five drug candidates were estimated to be effective in treating MPB. The lack of alpha-linolenic acid, an omega-3 fatty acid, can lead to scalp hemorrhagic folliculitis and maintaining adequate levels of alpha-linolenic acid can help strengthen the skin barrier [88–91]. Treatment with linolenic acid has been found to increase hair cell growth by antagonizing Wnt/β-catenin signaling, making it effective for treating baldness [92]. Estrogen-related drugs such as afimoxifene, diethylstilbestrol, and genistein have been reported to alleviate hair loss by controlling abnormal estrogen levels [93–96]. Succinic-acid, used in ATP generation reaction in the mitochondria, can promote hair growth by increasing ATP concentration in the scalp and regulating the inflammatory response [58, 97–99]. In MPB type 3, the MPB signatures were involved in cellular functions such as transcriptional processes and chromosomal remodeling, which may cause drug-induced cell death when targeted [100]. 

While our study has provided insights into the complex underlying mechanisms of MPB, there are still some limitations within the study design. First, there was no scalp skin panel in GTEx, so we had to use available skin panels obtained from the suprapubic or lower leg instead of scalp skin. However, it is important to note that the scalp belongs to the ectoderm-oriented epidermis, thus choosing skin panels was the best alternative for this study. Although MPB is associated with sex chromosomes, the GWAS datasets we used only include autosomal chromosomes. This was done intentionally to observe the effects of MPB on autosomal chromosomes only in this study. Additionally, since this study was performed *in silico* approach, our findings need to be validated with additional experimental studies. Especially, some drug candidates for treating MPB have not been studied yet, so the efficacy and delivery method of these drug candidates need to be further tested using in vivo and in vitro models. Despite these limitations, we believe that our study will contribute to the understanding of the genetic mechanisms of MPB and provide new insights for treatment of MPB.

### Electronic supplementary material

Below is the link to the electronic supplementary material.

Supplementary Material 1

Supplementary Material 2

Supplementary Material 3

## Data Availability

The GWAS summary statistics used in this study can be found in GWAS Atlas (https://atlas.ctglab.nl/) with the accession ID 3501, 3502, and 3503. Multi-tissue gene expression and chromatin modification datasets for LDSC-SEG can be downloaded in following github page (https://github.com/bulik/ldsc/wiki/Cell-type-specific-analyses). Tissue-specific eQTL panels from GTEx can be downloaded from FUSION (http://gusevlab.org/projects/fusion/). Tissue-shared eQTL panels from CONTETN can be found in following github page (https://github.com/cozygene/CONTENT). Functional gene sets can be retrieved from MsigDB (http://software.broadinstitute.org/gsea/msigdb). Previously reported MPB markers were searched on Open Targets Platform (https://platform.opentargets.org/). All data generated during this study are included in this published article and its supplementary files.

## Abbreviations

*ADAMTS18*: *ADAM Metallopeptidase with Thrombospondin Type 1 Motif 18*
*AR*: *Androgen Receptor*
*ATP6V0D1*: *ATPase H + Transporting V0 Subunit D1*
*CD59*: *Cluster of Differentiation 59*
*CHD6*: *Chromodomain Helicase DNA Binding Protein 6*
CLUE: Connectivity Map and Library of Integrated Network-based Cellular Signatures Unified Environment
CMAP: Connectivity Map
COLOC: Colocalization
CONTENT: Context-specific Genetics
ENCODE: Encyclopedia of DNA Elements
eQTL: Expression Quantitative Trait Loci
*FADS1*: *Fatty Acid Desaturase 1*
FUSION: Functional Summary-based Imputation
GO: Gene Ontology
GSEA: Gene Set Enrichment Analysis
GTEx: Genotype-tissue Expression
GWAS: Genome-wide Association Study
HNS: Hamilton-Norwood Scale
*KANSL1*: *KAT8 Regulatory NSL Complex Subunit 1*
LD: Linkage Disequilibrium
LDSC: LD Score Regression
LDSC-SEG: Linkage Disequilibrium Score-specifically Expressed Genes
LINCS: Library of Integrated Network-based Cellular Signatures
*MAPT*: *Microtubule-associated Protein tau*
MPB: Male-pattern baldness
NES: Normalized Enrichment Scores
PheWAS: Phenome-wide Association Study
PP: Posterior Probabilities
PPI: Protein-protein Interaction
SNPs: Single Nucleotide Polymorphisms
*TMEM178B*: *Transmembrane Protein 178B*
TWAS: Transcriptome-wide Association Studies
UKBB: UK biobank
*ZDHHC5*: *Zinc Finger DHHC-type Palmitoyltransferase 5*
*ZIC2*: *Zic Family Member 2*
