# Supplementary material for: Prioritizing susceptibility genes for the prognosis of male-pattern baldness with transcriptome-wide association study
Source: Hum Genomics. 2024 Apr 2;18:34. doi: 10.1186/s40246-024-00591-y (PMC10985920; doi:10.1186/s40246-024-00591-y)
Supplement: Supplementary file 3 — Supplementary Material 3 [file 40246_2024_591_MOESM3_ESM.docx]

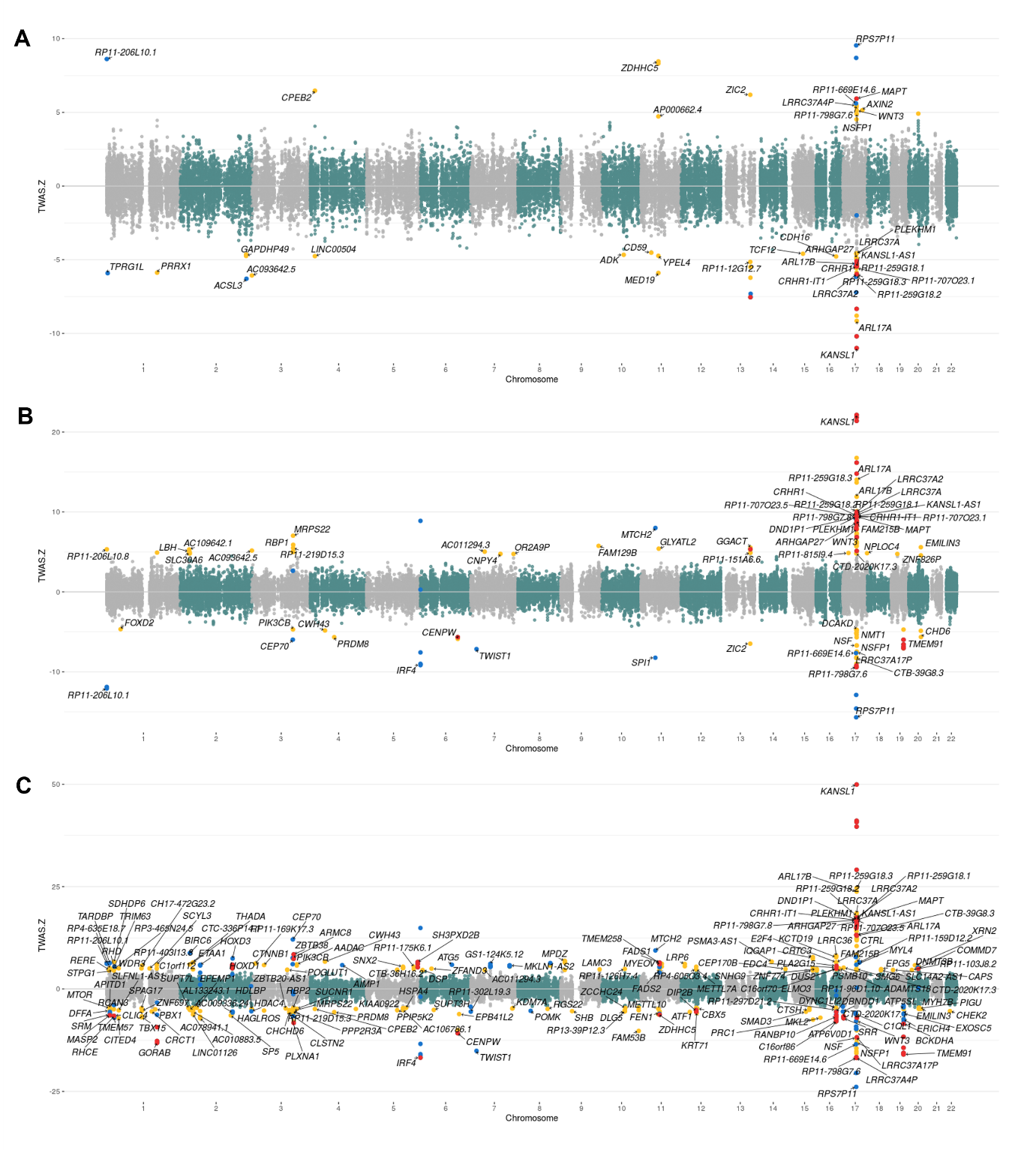


**Supplementary figure 1. Results of TWAS using GTEx and CONTENT panels.** Manhattan plots presenting TWAS results of (A) MPB type 2, (B) type 3, and (C) type 4. The X-axis represents the chromosomal position of the genes, while the Y-axis represents the TWAS z-score values obtained from the predicted expression level of each gene. The yellow dots indicate significant associations in the results of GTEx single-tissue panels, while the blue dots indicate significant associations in the results of CONTENT cross-tissue panels. The red dots indicate significant associations in both single-tissue and cross-tissue panels. If associations were identified in multiple tissue panels simultaneously, the gene names were presented based on the largest absolute TWAS z-score.

**
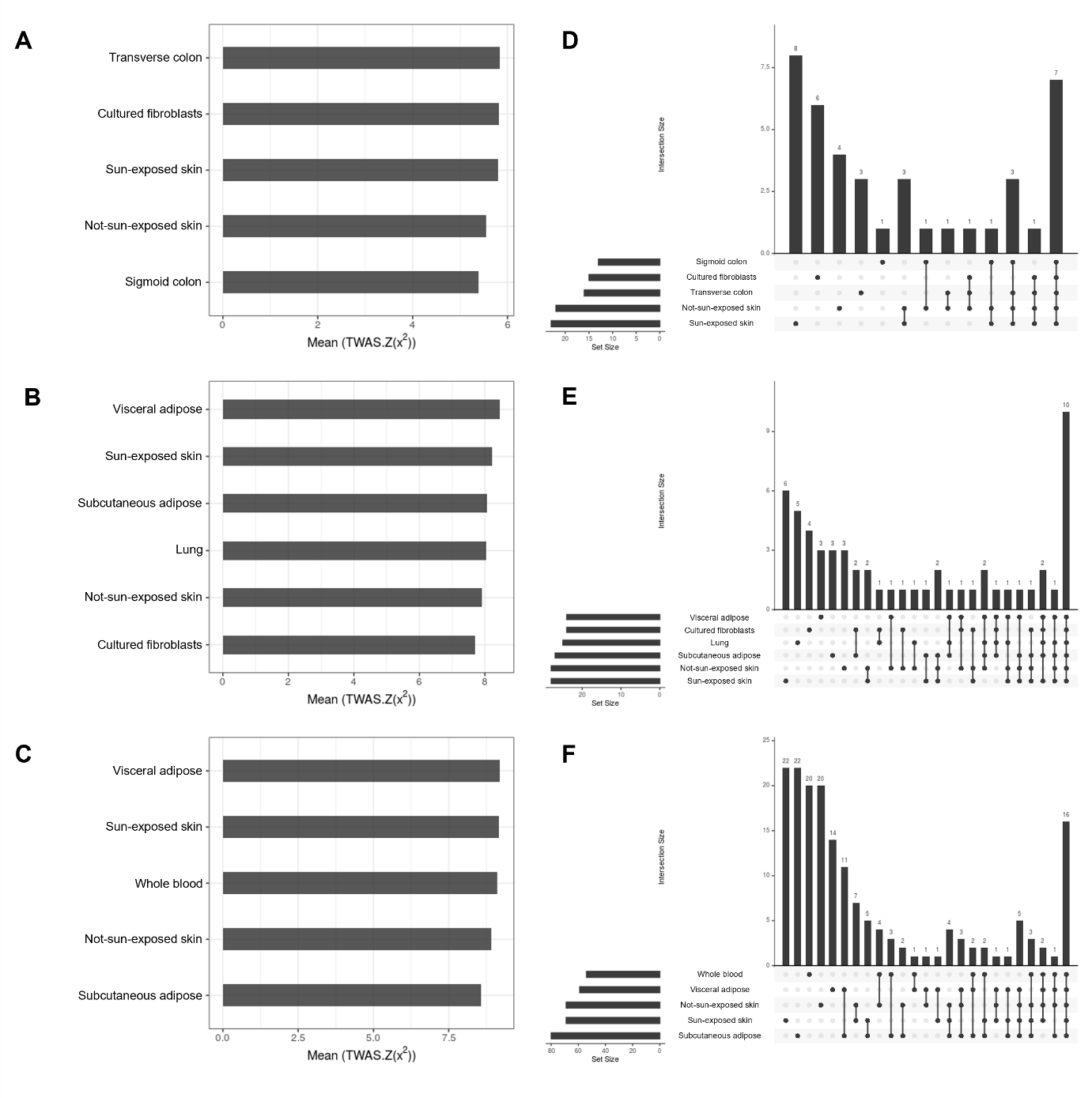
**

**Supplementary figure 2. The comparison of results obtained from GTEx panels.** The tissue-specific effects of TWAS results with GTEx panels were analyzed by calculating the mean of squared Z-score for each tissue of (A) MPB type 2, (B) type 3, and (C) type 4. Upset plots showing the number of gene intersections for each tissue of GTEx in (D) MPB type 2, (E) type 3, and (F) type 4.

**
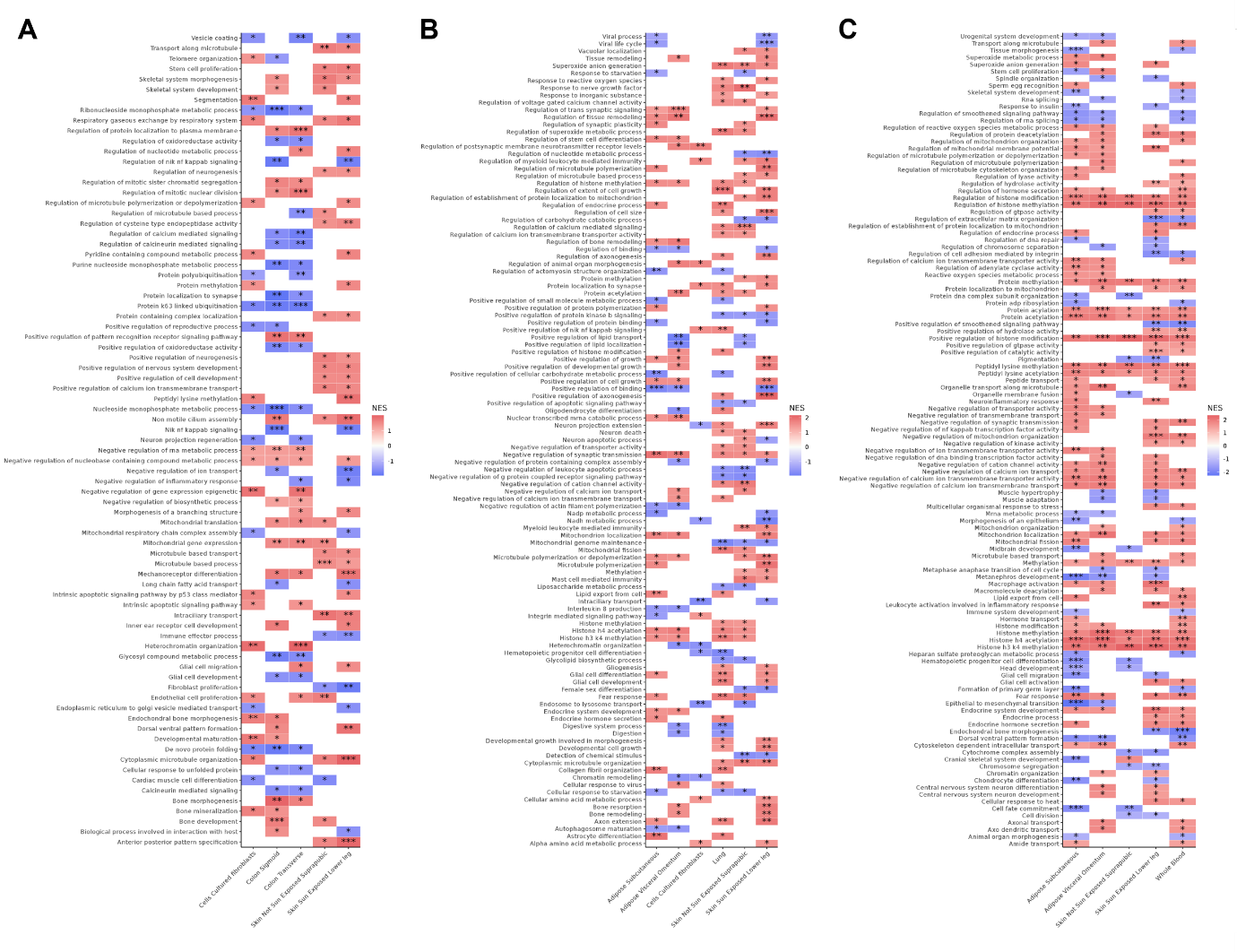
**

**Supplementary figure 3. Results of GSEA for individual tissue panels of GTEx.** Heatmaps showing the pathways enriched in multiple tissue panels for (A) MPB type 2, (B) type 3, and (C) type 4. The color reflects NES, indicating the degree of up-regulated and down-regulated gene enrichment (*: P-value < 0.05; **: P-value < 0.01; ***: P-value < 0.001).

**
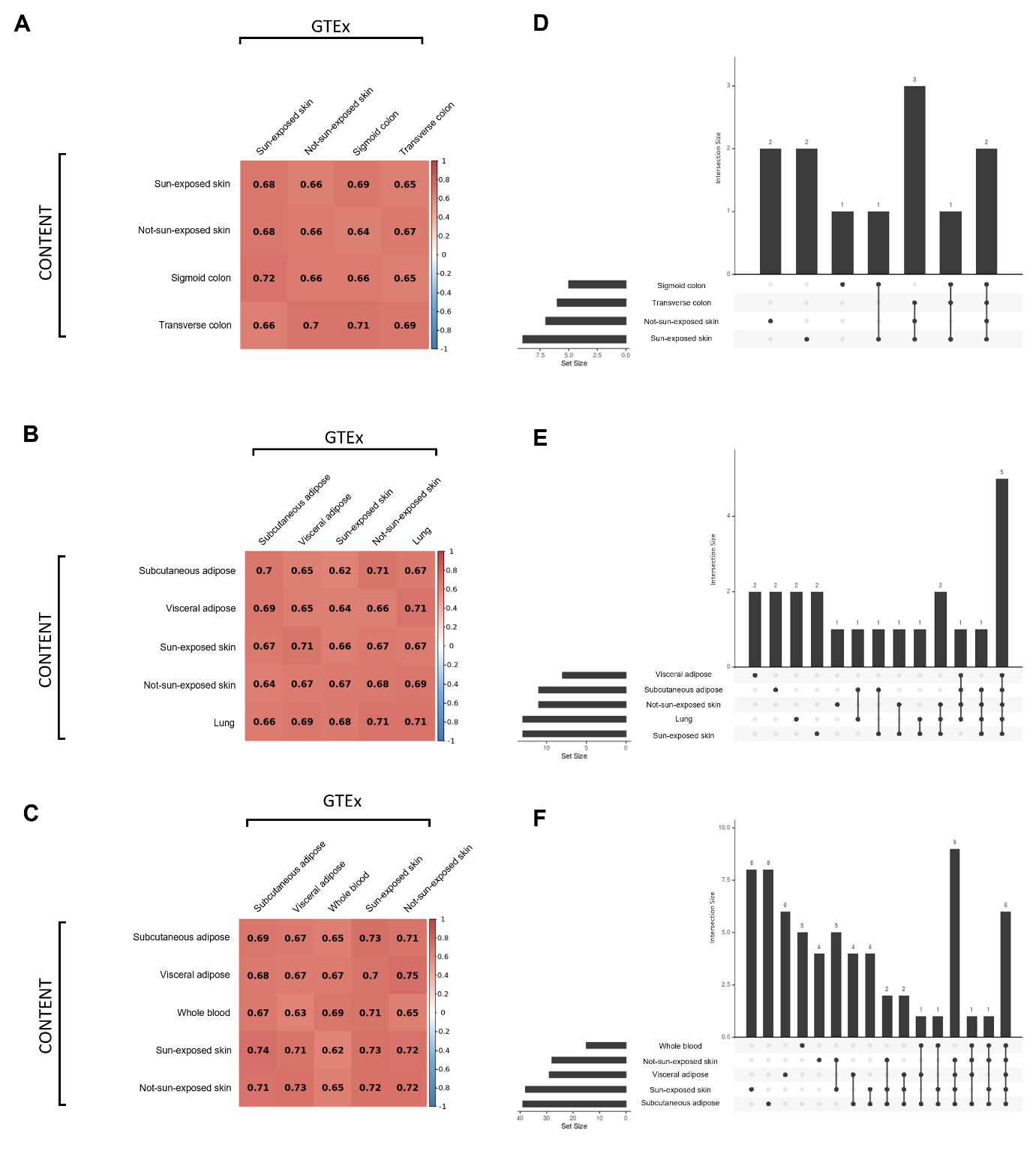
**

**Supplementary figure 4. The comparison of results from CONTENT panels.** Heatmaps showing the correlation between the results of GTEx and CONTENT panels in (A) MPB type 2, (B) type 3, and (C) type 4. Upset plots displaying the number of gene intersections for each tissue of CONTENT in (D) MPB type 2, (E) type 3, and (F) type 4.

**
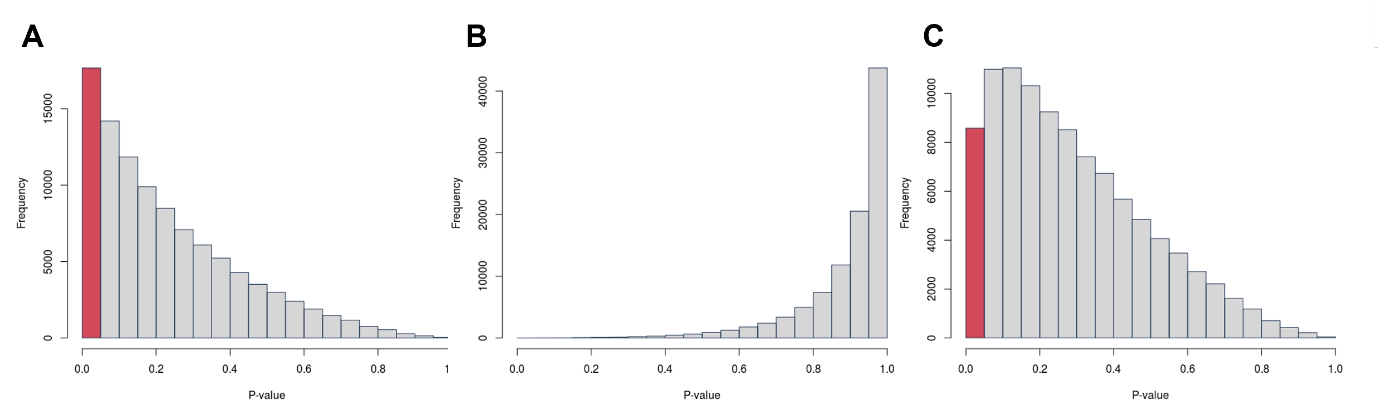
**

**Supplementary figure 5. The results of the gene interaction analysis using the PPI score.** The bar plots representing the P-value of the interaction score between known MPB genes and MPB signatures. This analysis was conducted using a one-tailed t-test with 10,000 repetitions for (A) MPB type 2, (B) type 3, and (C) type 4.
